# *Sclerotinia* Species Causing Lettuce Drop Disease in Serbia

**DOI:** 10.3390/microorganisms14010189

**Published:** 2026-01-14

**Authors:** Maja Živanović, Milica Mihajlović, Aleksandra Jovanović, Jovana Hrustić, Mira Vojvodić, Brankica Pešić, Aleksandra Bulajić

**Affiliations:** 1 Institute of Pesticides and Environmental Protection, Banatska 31b, 11080 Belgrade, Serbia; maja.zivanovic@pesting.org.rs (M.Ž.); milica.mihajlovic@pesting.org.rs (M.M.); jovana.hrustic@pesting.org.rs (J.H.); brankica.pesic@pesting.org.rs (B.P.); 2 Institute for the Application of Nuclear Energy INEP, University of Belgrade, Banatska 31b, 11080 Belgrade, Serbia; ajovanovic@inep.co.rs; 3 Faculty of Agriculture, University of Belgrade, Nemanjina 6, 11080 Belgrade, Serbia; miravojvodic2510@gmail.com

**Keywords:** *Lactuca sativa*, morphological characterization, pathogenicity, virulence, phylogeny, *Sclerotinia sclerotiorum*, *Sclerotinia minor*, oxalic acid

## Abstract

*Sclerotinia* spp. are globally distributed, devastating plant pathogens with a broad host range, including lettuce, on which they cause lettuce drop disease. To investigate the geographical distribution of lettuce drop incidence and the population structure of *Sclerotinia sclerotiorum* and *S. minor* in Serbia, 27 commercial lettuce fields across 12 administrative districts were surveyed. *Sclerotinia* spp. were confirmed at 10 localities, with *S. sclerotiorum* occurring more frequently. Co-occurrence of both species within the same field was recorded at only one location. Clear phenotypic and physiological differences were found between *Sclerotinia* species, as well as among isolates within each species. The two species differed in colony appearance, sclerotia production, virulence, growth rate, oxalic acid production, and tolerance to elevated osmotic pressure. Haplotype analysis of *S. minor* revealed the existence of 9 haplotypes arranged in a star-shaped network. These findings highlight the importance of considering both inter- and intraspecific variability of *Sclerotinia* species when evaluating their impact on crops, improving our understanding of *Sclerotinia* populations in lettuce, and supporting the development of effective management strategies.

## 1. Introduction

Lettuce (*Lactuca sativa* L.) is an important leafy vegetable cultivated extensively in both open-field and protected environments. Global lettuce production exceeded 28 million metric tons in 2023, with major producers including China, the United States, India, and Spain. Besides Spain, Italy, Turkey, and Belgium are the major lettuce-producing countries in Europe (FAO, https://www.fao.org/faostat/en/#data/QCL, accessed on 10 August 2025). Official data on lettuce production in Serbia are currently unavailable, despite being one of the most extensively cultivated vegetable crops in the country [1].

A wide spectrum of fungal and oomycete pathogens poses a persistent threat to lettuce production, often resulting in substantial reductions in yield and postharvest quality. Reported yield losses range from 5% to complete crop failure, depending on the pathogen and the disease incidence [2,3,4]. Lettuce drop, also known as Sclerotinia rot, is among the most economically significant diseases worldwide, affecting all types of lettuce, particularly leaf, romaine, and head lettuce [5,6]. While diseases caused by *Sclerotinia* species were typically sporadic in crop production, they have become increasingly persistent under intensive farming systems characterized by short crop rotations [7]. Yield losses in field-grown lettuce in the United Kingdom average around 10%, but can reach up to 50% under conditions of heavy or sustained precipitation [2]. Reported losses in Australia’s major lettuce-producing areas were between 10% and 45%, even when regular fungicide spray programs were implemented [8].

Two species of the genus *Sclerotinia*, *S. sclerotiorum* and *S. minor*, are known as the causal agents of Sclerotinia rot on lettuce and other composite vegetables [9,10]. The third lettuce drop causal agent, *S. nivalis* was recently isolated and identified in China [11]. Overall, *S. sclerotiorum* is one of the most devastating soilborne plant pathogens [12,13]. It is distributed in all continents, in more than 92 countries (CABI distribution map, UK, https://plantwiseplusknowledgebank.org, accessed on 10 August 2025), and has an extraordinarily broad host range, infecting more than 450 plant species from more than 75 families [9,13]. The hosts are mostly dicotyledonous herbaceous plants, although several monocotyledonous species were also recorded. *S. minor* is the most economically important in lettuce, sunflower, green bean, and peanut, even though it could cause substantial losses in other crops, especially if rotated with lettuce. They cause almost identical symptoms but have significantly different dispersal patterns and modes of infection [14].

Understanding the pathogenic potential and ecological success of *Sclerotinia* spp. requires an integrated assessment of their physiological traits and population structure. Oxalic acid production is a key virulence factor that facilitates host colonization through tissue acidification, suppression of plant defense responses, and cell wall degradation [15,16]; however, its quantitative contribution to pathogenicity may vary among species and isolates [12,17]. Tolerance to elevated osmotic pressure represents an important adaptive trait of soilborne pathogens, reflecting their ability to persist and grow under adverse environmental conditions such as high soil salinity or limited water availability, and may influence both soil survival and competitiveness during host infection [18]. In parallel, haplotype analysis provides insight into population genetic structure and diversity by revealing dominant lineages and patterns of genetic relatedness. A high degree of genetic diversity can also indicate a greater ability to adapt to changing environmental conditions and may reflect increased biological fitness of these genotypes [19]. Together, studies on haplotype composition, oxalic acid production, and osmotic stress tolerance provide a framework for exploring potential associations between population genetic structure and variation in key functional traits in *Sclerotinia* spp. associated with lettuce drop disease. 

Data on the distribution and impact of *Sclerotinia* species in Serbia, particularly on leafy vegetables including lettuce, are scarce [20]. The only available record is a recent detection of *S. minor* as a lettuce pathogen [21], while there are no data on *S. sclerotiorum* as a lettuce pathogen. Serbian lettuce producers have expressed increasing concern and frequently requested management advice regarding lettuce drop disease. Therefore, a study was conducted to identify the distribution and species composition of *Sclerotinia* spp. causing lettuce drop disease in Serbia. The specific objectives of this study were to (1) identify *Sclerotinia* species associated with symptomatic lettuce plants in Serbia using conventional morphological and molecular methods; (2) assess the relative abundance and distribution of each *Sclerotinia* species across different lettuce-growing regions in Serbia; (3) characterize selected isolates of *Sclerotinia* spp. with respect to their virulence and morphological traits; (4) evaluate genetic diversity of the isolates through phylogenetic analyses and haplotype assessment; (5) examine physiological properties of the isolates such as growth rate and oxalic acid production and (6) determine the susceptibility of the isolates to high osmotic pressure as an indicator of their physiological adaptability to the environment.

## 2. Materials and Methods

### 2.1. Sclerotinia spp. Isolates

To assess the presence and spatial distribution of lettuce drop in Serbia, field surveys were conducted in the spring seasons of 2021 and 2022. Symptomatic lettuce plants, exhibiting poor or stunting growth and blanching of leaves, were collected from 27 commercial unheated plastic tunnel fields in 12 administrative districts of Serbia. The disease incidence was estimated by walking through the crop in a zigzag pattern and randomly rating 100 plants in three replicates. The number of samples collected per site was estimated based on field size (ranging from 0.2 to 2 ha) and the disease incidence and severity. Symptomatic tissues from sampled plants were cut into small pieces, surface sterilized with 0.5% sodium hypochlorite for 1 min, rinsed three times in sterile distilled water, placed on potato dextrose agar (PDA) supplemented with streptomycin (300 mg/mL), and incubated for 4–7 days at 25 °C. From the emerging fungal colonies, monohyphal-tip isolates were derived. The obtained isolates were stored at −80 °C in 20% glycerol for long-term preservation, while short-term storage was maintained on PDA slants at 4 °C.

All the obtained isolates were preliminarily identified based on colony morphology, growth rate, and the number and size of produced sclerotia after the incubation of 15 days on PDA medium at 25 °C in darkness [22].

### 2.2. Pathogenicity Test

To confirm the pathogenicity of all the obtained *Sclerotinia* spp. isolates, artificial inoculation of 4-leaf-stage lettuce plants cv. Majska kraljica, grown on commercial growth substrate (Floragard, Oldenburg, Germany) in 1 L pots, was conducted. Mycelial plugs, 10 mm in diameter, were excised from the margin of a 3-day-old colony grown on PDA and placed mycelium-side down on undamaged ground-level leaves of lettuce plants. Five plants were inoculated per isolate. Control plants were inoculated with sterile PDA plugs. Inoculated plants were enclosed in transparent plastic bags to maintain high humidity, and the bags were misted internally with water. Plants were kept in a growth chamber at 20 °C under a 13 h photoperiod. Plants were monitored daily for symptom development. Following symptom appearance, the pathogen was reisolated, and the morphological characteristics of the recovered isolates were compared with those of the challenging isolates.

### 2.3. Molecular Detection of Sclerotinia spp. Isolates

Preliminary identification of all obtained *Sclerotinia*-like isolates was confirmed with multiplex polymerase chain reaction (PCR) and respective specific primers. Total genomic DNA was isolated from mycelia of 7-day-old cultures grown on PDA using a method described by Harrington and Wingfield [23]. Primer pairs SMLcc2F/SMLcc2R, specific for *S. minor*, SSasprF/SSasprR for *S. sclerotiorum,* and STCadF/STCadR for *S. trifoliorum*, were used for amplifications of the Lcc2, Aspr, and Cad gene regions, respectively [24]. The multiplex PCR mix contained 12.5 μL of 2× Master mix (Fermentas, Vilnius, Lithuania), 1 μL of each primer, 1 μL of template DNA, and molecular-grade water up to a final volume of 25 μL. PCRs were performed in a Biometra Thermocycler (Analytik Jena GmbH+Co., Jena, Germany) with the following reaction conditions: an initial denaturation at 95 °C for 3 min, followed by 35 cycles of 95 °C for 30 s, 60 °C for 90 s, 72 °C for 90 s, and a final extension at 72 °C for 7 min. Negative controls were included by replacing template DNA with molecular-grade water. The PCR products were separated by electrophoresis in 2% agarose gels run in 1× Tris-borate EDTA buffer at 100 V constant voltage. The gels were stained with ethidium bromide, and the products were visualized and photographed under ultraviolet (UV) light.

### 2.4. Morphological Characterization

Based on geographic origin and colony morphology on PDA, 10 representative *S. sclerotiorum* and 10 *S. minor* isolates were selected for detailed phenotypic characterization. After 15 days of incubation on PDA at 25 °C in darkness, colony color, texture, and growth pattern were assessed. The presence, number, size, shape, and distribution of sclerotia within the Petri plate were also recorded [22]. To determine sclerotial size (length and width) 30 randomly selected sclerotia per isolate were measured using a ruler. Colony growth rate was determined after 2-day incubation on PDA in darkness and expressed in mm per day, following the methodology described by Morral et al. [25]. To study pigment production, isolates were incubated on PDA at 25 °C for 15 days, after which pigmentation was assessed by visually inspecting the reverse side of the plates [26]. All experiments were conducted twice, with four replicates per isolate.

### 2.5. Sequencing of Ribosomal DNA Internal Transcribed Spacer Region

The identity of selected representative *Sclerotinia* spp. isolates was further confirmed by amplification and sequencing of the internal transcribed spacer (ITS) region of ribosomal DNA (rDNA) using primers ITS1 and ITS4 and the same reaction mixture as described above. PCR amplifications were performed with an initial denaturation for 90 s at 94 °C, followed by 29 cycles consisting of a denaturation step for 30 s at 94 °C, primer annealing for 30 s at 55 °C, and extension for 30 s at 72 °C. The final extension step was performed at 72 °C for 9 min 30 s [27].

The amplified products were sequenced directly on automated equipment (Macrogen Inc., Seoul, Republic of Korea) in both directions using the same primers as for amplification. For each isolate, the consensus sequence covering the partial rDNA-ITS region was reconstructed using Pregap4 and Gap4 (v. 1.5) from the Staden Package and aligned using Clustal X under MEGA software version 6. All isolates were compared to all publicly available sequences using the Basic Local Alignment Search Tool (BLAST, version 2.13.0) algorithm in the National Center for Biotechnology Information (NCBI) database. The obtained sequences of Serbian *Sclerotinia* spp. isolates were deposited in the GenBank database, and their accession numbers were assigned.

### 2.6. Phylogenetic Analysis

A phylogenetic tree was reconstructed based on 20 ITS sequences generated in this study and 42 previously published sequences representing *S. sclerotiorum*, *S. minor*, *S. nivalis*, and *S. trifoliorum* isolates from different hosts and geographic origins retrieved from GenBank (Table 1) using the Maximum Parsimony algorithm implemented in MEGA 6. The tree was evaluated with 1000 bootstrap replications to test clade stability, and bootstrap values < 50% were omitted. The sequence of *Hypocrea lixii* (GenBank Acc. No. FJ861393) was used as an outgroup reference species.

### 2.7. Haplotype Analysis of S. minor Sequences

As the haplotype analysis of *S. sclerotiorum* was recently performed [29], the present study was focused on *S. minor* population. The haplotype analyses of *S. minor* was based on all 99 available ITS region sequences in GenBank (accessed on 10 January 2025), along with 10 Serbian *S. minor* sequences. After a manual review and exclusion of short and/or sequences with degenerate codons, a total of 103 sequences were included in the analysis. All sequences were compared by calculating nucleotide identities (nt) using MEGA X software [32]. The number of haplotypes (h), the haplotype diversity (Hd), the identification of polymorphic sites (S) and the nucleotide diversity (P) of the ITS region were analyzed using DnaSP version 6.0 [33]. Haplotype composition and frequency were further investigated using PopART software version 1.7 (Population Analysis with Reticulate Trees) [34]. The mutual genealogical relationships among haplotypes were visualized using the Median Joining Network algorithm implemented in PopART software [35].

### 2.8. Virulence Test and Oxalic Acid Quantification

The virulence of 10 *S. sclerotiorum* and 10 *S. minor* isolates was assessed using a detached leaf inoculation assay [36]. Fully expanded, healthy lettuce leaves were used for inoculation. Leaves were arranged in plastic trays (12 × 20 cm) with two layers of moist blotter paper placed at the bottom to maintain humidity. Mycelial discs (10 mm in diameter) were excised from the actively growing margins of 72 h cultures of *S. sclerotiorum* and *S. minor* and placed on the upper side near the edge of each leaf. Leaves inoculated with sterile PDA plugs of the same size served as a control. Trays containing the inoculated leaves were incubated in a growth chamber set at 25 °C, 85–90% relative humidity, and a 10/14 h day/night photoperiod. Lesion length (in mm) along the main vein of the inoculated leaves was measured 72 h post-inoculation. A completely randomized design (CRD) was employed with three replicates per isolate. The experiment was conducted twice to ensure reproducibility.

Oxalic acid production by the selected 10 *S. sclerotiorum* and 10 *S. minor* isolates was quantified and compared [37]. The isolates were grown statically in flasks containing 50 mL of potato dextrose broth (PDB) (2% glucose and 0.4% fresh potato extract in distilled water) with three replicates per isolate. After a 3-day incubation at 22 ± 2 °C, the cultures were vacuum-filtered, and the dry weight of the mycelial fraction was determined after drying at 80 °C for 72 h. For oxalic acid quantification, supernatant (200 µL), bromophenol blue (BPB, 1 mM, 110 µL) (Centrohem, Stara Pazova, Serbia), sulfuric acid (1 M, 198 µL) (Sigma-Aldrich, Schnelldorf, Germany), potassium dichromate (100 mM, 176 µL) (Merck, Darmstadt, Germany) and distilled water (4.8 mL) were mixed and incubated at 60 °C for 10 min. Subsequently, the reaction was quenched by sodium hydroxide solution (0.75 M, 500 µL) (Fisher Scientific, Ottawa, ON, Canada). All absorbance readings were performed in triplicate at 600 nm using a Shimadzu UV-1800 UV/Visible scanning spectrophotometer (Shimadzu, Kyoto, Japan), with sterile PDB as the blank control. The calibration curve was prepared using known concentrations of oxalic acid (Oleohemija, Belgrade, Serbia), and the results were expressed as μg oxalic acid per mg of mycelial dry weight.

### 2.9. Susceptibility of Sclerotinia spp. Isolates to High Osmotic Pressure

The susceptibility of 10 representative *S. sclerotiorum* and 10 *S. minor* isolates to high osmotic pressure was studied according to a slightly modified method of Beever and Brien [38]. Sodium chloride (NaCl) was added to PDA to achieve a final concentration of 0.51 mol/dm^3^, while PDA amended with sterile distilled water served as a control. Mycelial plugs (10 mm in diameter) were excised from the actively growing margins of 3-day-old colonies and placed, mycelium side down, at the center of Petri plates. The plates were incubated at 25 °C, and the mycelial growth diameter was measured in two perpendicular directions after 2 days, excluding the diameter of the inoculation plug. The percentage of growth inhibition under osmotic stress was calculated relative to the control. The experiment was conducted twice, with four replicates per treatment.

### 2.10. Statistical Analyses

To evaluate differences both between *S. sclerotiorum* and *S. minor* species and among isolates within each species, the data on mycelial growth, lesion diameters on lettuce leaves (virulence assay), and oxalic acid production in PDB were subjected to statistical analysis using GraphPad Prism version 5.0 (GraphPad Software, Boston, MA, USA). Statistical significance was set at *p* < 0.05. To compare the two species, an unpaired two-tailed *t*-test was used. Prior to the *t*-test, data normality was assessed using the Shapiro–Wilk test, and homogeneity of variances was evaluated using the F-test. Where significant differences in variances were detected, Welch’s correction was applied. Differences among isolates within each species were analyzed separately using the non-parametric Kruskal–Wallis test, followed by Dunn’s multiple-comparison test. Relationships between virulence and the other studied variables (growth rate, oxalic acid production, and susceptibility to high osmotic pressure) were examined using Spearman’s rank correlation coefficient.

## 3. Results

### 3.1. Presence and Distribution of Sclerotinia spp. in Serbia

**Figure 1:**
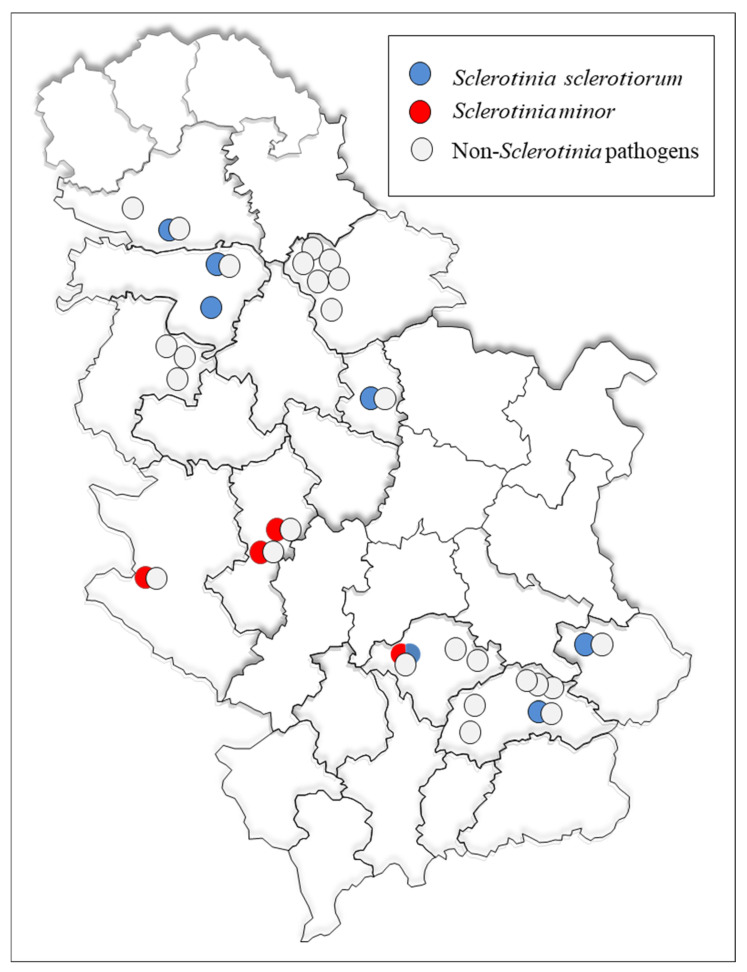
The geographic distribution of localities in Serbia included in the survey and detected *Sclerotinia* spp. species.

**Figure 2:**
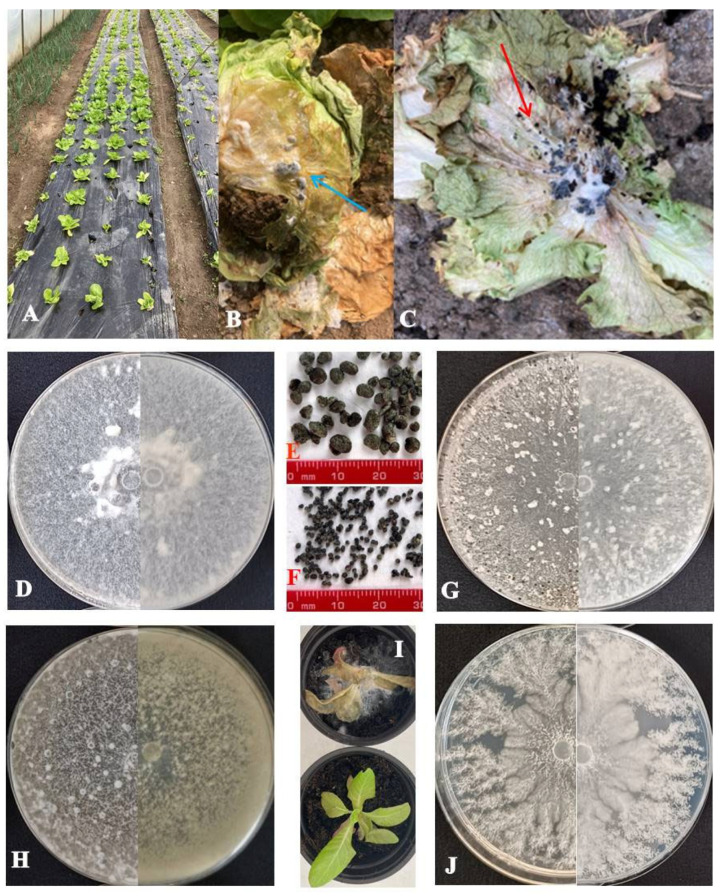
*Sclerotinia* spp.—naturally infected lettuce plants with *S. minor* (**A**,**C**) and *S. sclerotiorum* (**B**) in the field with developed sclerotia (arrows); obverse (left) and reverse (right) appearance of snow-white (**D**) and gray (**H**) mycelium of *S. sclerotiorum* isolates on potato dextrose agar (PDA) after 15 days at 25 °C; obverse (left) and reverse (right) appearance of regular (**G**) and irregular (**J**) growth pattern of *S. minor* isolates on PDA after 15 days at 25 °C; Sclerotia of *S. sclerotiorum* (**E**) and *S. minor* (**F**) isolates; (**I**) pathogenicity test: inoculated plant (top) and control (bottom).

At 27 sites distributed across 12 administrative districts in the major lettuce-producing regions of Serbia (Figure 1, Table 2), lettuce plants exhibiting symptoms of wilting, poor growth, stunting, blanching, and leaf decay (Figure 2A–C) were collected. From the collected samples, 78 *Sclerotinia*-like colonies were recovered, initially with uniform colony appearance, but with visible differences between two groups of isolates after 5–6 days of incubation. The obtained isolates forming large sclerotia were preliminarily identified as *S. sclerotiorum* (43 out of 78), whereas the isolates with numerous small sclerotia were assigned as *S. minor* (35 out of 78). Using the species-specific primers, products of predicted size of 171 bp for *S. sclerotiorum* and 264 bp for *S. minor* isolates were amplified, confirming the identification of all isolates. Based on morphology and pathogenicity tests, besides *Sclerotinia* spp., as the most frequently isolated pathogens, various fungal/pseudofungal species, from genera *Phoma*, *Verticillium*, *Alternaria*, *Plectosphaerella*, *Fusarium*, *Pythium*, and *Botrytis*, were detected.

In the fields where *Sclerotinia* species were detected, the estimated incidence of lettuce drop ranged from 2% to 55%, with a mean value of 25.7% (Table 2). The highest incidence (55%) was recorded at the Potočanje locality, where symptoms included extensive leaf necrosis and the development of abundant white mycelium and numerous sclerotia on infected plants. In the other surveyed localities, the disease incidence was lower, ranging from 2% in Kraljevci (Srem District) to 40% in Irig (Srem District). *S. sclerotiorum* occurred at 7 locations in 6 districts, while *S. minor* was detected at 4 locations in 3 districts. Co-occurrence of both species was observed only at the Blace locality in the Toplica District (Figure 1, Table 2).

### 3.2. Pathogenicity 

The pathogenicity of all 78 obtained *Sclerotinia* spp. isolates was confirmed by artificial inoculation of lettuce plants. After 3–7 days, all inoculated plants exhibited characteristic lettuce drop symptoms, while control plants remained asymptomatic (Figure 2I). No differences were observed in the timing of symptom appearance, nor in their severity. The pathogen was reisolated from symptomatic tissue following the previously described method, whereas no isolates were obtained from control plants. Koch’s postulates were fulfilled by verifying the identity of the reisolates based on their morphological characteristics.

### 3.3. Morphological Characterization of Sclerotinia spp. Isolates

Ten representative *S. sclerotiorum* isolates produced between 2.3 and 65 large sclerotia per plate, with diameters ranging from 0.67 to 5.83 mm (Figure 2E). Colony color varied from snow white (20% of isolates) to white (30%), whitish (40%), and 10% gray, as observed in isolate Ss 18-10 (Figure 2D,H). Mycelial density ranged from sparse to very dense, exhibiting textures described as floccose, woolly, or woolly-floccose. Most isolates lacked dark pigmentation, with the exception of Ss 18-10 and Ss 20-3, which displayed noticeable pigmentation. *S. minor* isolates produced 11.7 to 373.3 small sclerotia per plate (Figure 2F), within the diameter range of 0.20–2.50 mm. Colonies were snow white (10%), white (10%), or whitish (80%), with floccose, cottony, or woolly mycelium of variable density. All isolates exhibited regular growth with even margins, except *S. minor* isolate Sm 18-5, which displayed irregular growth characterized by a lobed margin (Figure 2G,J). Patterns of sclerotial distribution also differed between species. *S. sclerotiorum* isolates most frequently had edge and middle ring sclerotia arrangements (50%) or scattered patterns (40%), with the middle ring pattern occurring rarely. In contrast, *S. minor* isolates displayed predominantly uniform sclerotial distribution, with the scattered pattern prevailing (80%) and the edge ring pattern restricted to the isolates Sm 18-4 and Sm 18-5. In terms of the time required for the formation of sclerotia, the difference was not observed between species. For both species, sclerotia mostly appeared on the eighth day of incubation on PDA medium at 25 °C. None of the *S. minor* isolates produced dark pigment, while pigment production in *S. sclerotiorum* isolates was limited to isolates Ss 18-10 and Ss 20-3 (Table 3).

**Figure 3:**
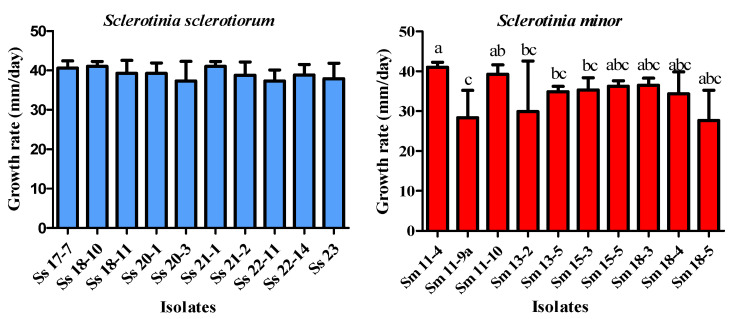
Growth rate of *Sclerotinia* spp. isolates grown on potato dextrose agar at 25 °C in the dark. The error bars represent the standard deviation. Values marked with the same letters or absence of letters indicate no statistically significant difference.

Colony growth rate varied significantly between species (t = 3.238, df = 10, *p* < 0.01). *S. sclerotiorum* exhibited a higher average growth (39.12 mm/day) compared to *S. minor* (34.19 mm/day), as it is presented in the Section 3.8. The difference in mycelial growth among *S. sclerotiorum* isolates was not significant (*p* = 0.0927), while the growth rate of *S. minor* isolates differed significantly (*p* < 0.01). For instance, a significant difference was observed between *S. minor* isolate Sm 11-4 and the isolates Sm 11-9a, Sm 13-2, Sm 13-5, and Sm 18-5. Differences were also observed between Sm 11-10 and Sm 18-5 (Figure 3).

### 3.4. Molecular Characterization and Phylogenetic Analysis

BLAST analysis showed that the ITS sequence of 10 Serbian *S. sclerotiorum* isolates had 100% nucleotide identity with GenBank *S. sclerotiorum* sequences, while sequences of 10 *S. minor* isolates had 100% nucleotide identity to *S. minor* from other parts of the world. Moreover, comparison of all ITS sequences obtained in this study revealed 100% nucleotide identity among isolates of the same species, and a difference of 3 nucleotides between *Sclerotinia* species.

**Figure 4:**
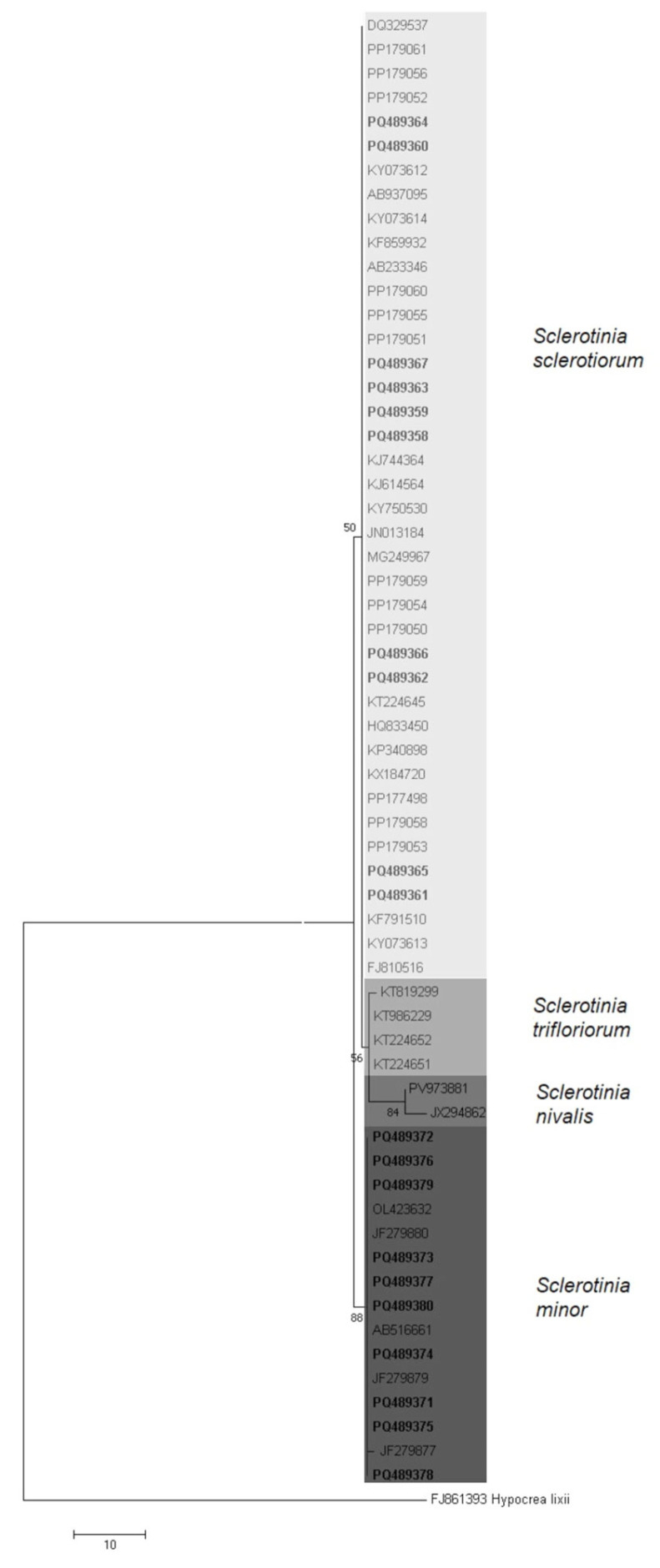
Maximum parsimony tree, constructed based on DNA sequences from fragments of the ITS rDNA from a subset of 20 Serbian isolates (10 *Sclerotinia sclerotiorum*, 10 *Sclerotinia minor*), 42 selected sequences of *Sclerotinia* species available in the GenBank database, and outgroup *Hypocrea lixii* (FJ861393). The tree was generated in MEGA 6. Bootstrap analyses were performed with 1000 replicates, and bootstrap values (>50%) are shown on the tree. All Serbian isolates are indicated in bold.

The phylogenetic analysis based on ITS rDNA sequences resulted in a well-supported tree consistent with the previously established relationships among *Sclerotinia* species (Figure 4). Two major clusters corresponding to *S. sclerotiorum* and *S. minor* were observed. Within the first cluster, *S. nivalis* and *S. trifoliorum* formed distinct subgroups closely related to *S. sclerotiorum*. Isolates of *S. sclerotiorum* from Serbia grouped with reference isolates from Asia, Europe, and North America, while *S. minor* isolates clustered with sequences from Asia, Europe, and Australia. These results confirm the molecular identification and phylogenetic placement of Serbian *Sclerotinia* isolates within the established global lineages.

### 3.5. Haplotype Structure of S. minor Sequences

**Figure 5:**
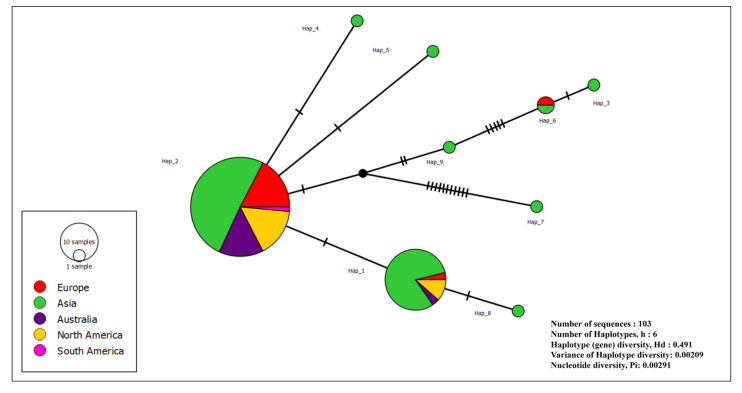
Haplotype network of *Sclerotinia minor* based on sequences of the ITS region (*n* = 103; 93 sequences from GenBank and 10 Serbian *S. minor* sequences). Each circle depicts a unique haplotype, and the size of each circle is proportional to the number of sequences it represents. Nucleotide differences are denoted by the hatch marks across black lines connecting haplotypes, with each hatch mark representing a single nucleotide variation.

The total of 103 *S. minor* sequences from Europe, Asia, Australia, North and South America showed the presence of 9 haplotypes (Hap1–9) with 22 variable positions. Hap2 was the most widespread, with 69 sequences from different continents and hosts, including all Serbian sequences. Hap1 was the second most common, with 26 isolates. The remaining haplotypes, Hap3–9, were less common, with 1 to 2 sequences. Haplotype diversity (Hd) was 0.491, indicating a moderate level of genetic variation within the population. Haplotype diversity variance was 0.00209. Nucleotide diversity (Pi) was 0.00291 (0 to 15 nt difference), indicating an overall low level of nucleotide variability among the sequences analyzed. The haplotype network of *S. minor* showed a structure with Hap2 in the center, encompassing all Serbian sequences (Figure 5). All other haplotypes were directly linked to Hap2 by single or multiple mutations.

### 3.6. Virulence of Sclerotinia spp. Isolates

After a 3-day incubation, all 20 selected isolates of both *Sclerotinia* species caused necrotic lesions on inoculated lettuce leaves, whereas none of the leaves inoculated with sterile agar plugs developed symptoms.

A statistically significant difference in virulence between the two species (t = 3.954, df = 12, *p* = 0.0019) was found, revealing that *S. sclerotiorum* is significantly more virulent under the tested conditions; an average lesion length caused by *S. sclerotiorum* was 46.64 mm, compared to 29.14 mm that was recorded for *S. minor*, as it is shown in the Section 3.8. 

**Figure 6:**
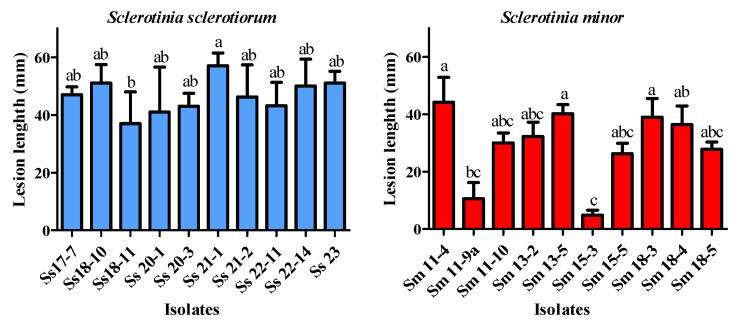
Lesion length on inoculated lettuce leaves caused by selected *Sclerotinia* spp. isolates after 3-day incubation at 25 °C. The error bars represent the standard deviation. Values marked with the same letters or absence of letters indicate no statistically significant difference.

The difference in virulence among isolates within both species was also observed (Figure 6). *S. sclerotiorum* isolate Ss 21-1 was highly virulent and caused lesions 57 mm long, while the isolate Ss 18-11 was the least virulent (lesion length 37 mm). The *S. minor* isolates formed significantly smaller lesions (4.8–44.2 mm). A positive correlation was recorded between virulence and the growth rate of *Sclerotinia* spp. (r = 0.709, *p* = 0.0005).

**Figure 7:**
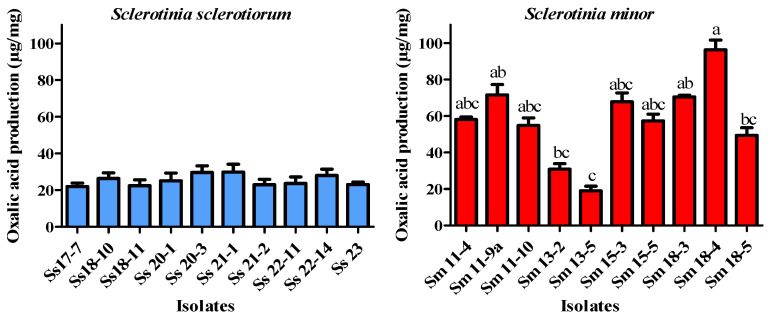
Oxalic acid production of different isolates of *Sclerotinia sclerotiorum* and *Sclerotinia minor* after 3-day static incubation in 50 mL of potato-dextrose broth at 25 °C. The error bars represent the standard deviation. Values marked with the same letters or absence of letters indicate no statistically significant difference.

Given that oxalic acid is a major virulence factor in *Sclerotinia* spp. and is strongly linked to fungal pathogenicity, oxalic acid production was assessed in parallel with pathogenicity assays to provide a comprehensive evaluation of the isolates’ virulence. Statistical analysis revealed that *S. sclerotiorum* produced significantly less oxalic acid than *S. minor* (t = 4.654, df = 9, *p* = 0.0012). The highest production was recorded in *S. minor* isolate Sm 18-4 (96.3 µg/mg), whereas the lowest was found in the isolate Sm 13-5 (19.0 µg/mg). The isolates of *S. sclerotiorum* exhibited lower levels of oxalic acid production. The highest amount was detected in isolate Ss 21-1 (29.9 µg/mg) (Figure 7). A statistically significant correlation between virulence and oxalic acid production in both *Sclerotinia* species was not found (*S. sclerotiorum*: r = 0.353, *p* = 0.313; *S. minor*: r = −0.188, *p* = 0.607).

### 3.7. High Osmotic Pressure Susceptibility 

**Figure 8:**
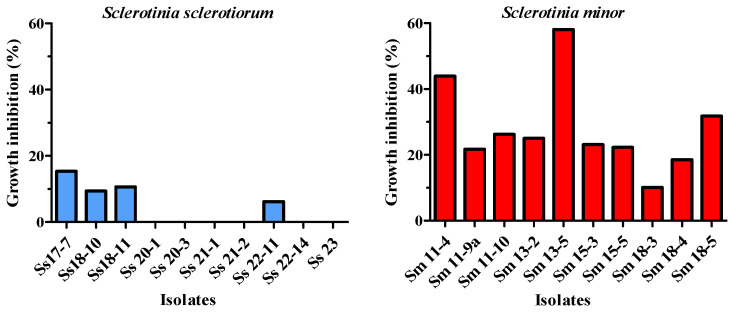
Susceptibility of *Sclerotinia sclerotiorum* and *Sclerotinia minor* isolates to high osmotic pressure: inhibition of the mycelial growth on potato dextrose agar medium supplemented with 0.51 mol/dm^3^ NaCl after incubation for 2 days at 25 °C.

*S. sclerotiorum* was significantly less susceptible to high osmotic pressure compared to *S. minor* (t = 4.859, df = 10, *p* = 0.0007). The growth of 60% of *S. sclerotiorum* isolates was not inhibited on a PDA medium supplemented with 0.51 mol/dm^3^ NaCl. The remaining 40% of the isolates showed growth inhibition ranging from 6.2% to 15.3%. In contrast, all *S. minor* isolates exhibited growth inhibition under the same conditions, ranging from 10.1% to 58.1% compared to the control (Figure 8). No statistically significant correlation was found between isolate susceptibility to increased osmotic pressure and virulence in either of the two studied species.

### 3.8. Comparative Characterization of S. sclerotiorum and S. minor

**Figure 9:**
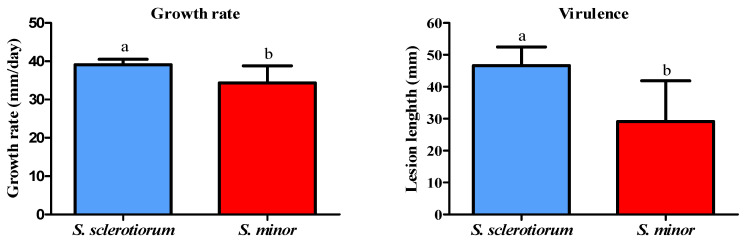

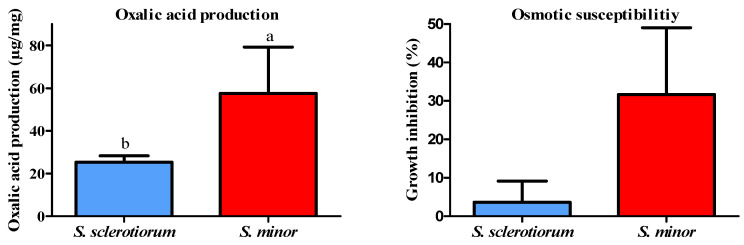
Comparison of *Sclerotinia sclerotiorum* and *Sclerotinia minor* in terms of the mycelial growth, virulence, oxalic acid production, and susceptibility to high osmotic pressure. An average of 10 isolates of each species is presented. The error bars represent the standard deviation. Values marked with different letters indicate statistically significant difference.

Based on the overall comparison of all examined traits, significant differences between *S. sclerotiorum* and *S. minor* were observed. Differences were detected in growth, virulence, oxalic acid production, and tolerance to high osmotic pressure. *S. sclerotiorum* was characterized by greater mycelial growth, higher virulence, and increased tolerance to elevated osmotic pressure. In contrast, *S. minor* was found to produce higher levels of oxalic acid (Figure 9).

## 4. Discussion

This study provides the first comprehensive characterization of *Sclerotinia* species as the causal agents of lettuce drop disease in Serbia. Two closely related broad-host-range pathogens, *S. sclerotiorum* and *S. minor* are detected, both described to infect lettuce and other members of the Asteraceae family [9,10]. *S. sclerotiorum* is a widespread pathogen of sunflower in Serbia, causing significant yield losses [39,40]. It was also detected in green beans [41], faba beans [42], and cabbage [29]. The presence of *S. minor* in Serbia was described based on symptom presence [43], with no additional data including pathogenicity confirmation. Recently, *S. minor* was isolated for the first time in Serbia from symptomatic lettuce plants [21]. *S. minor* could become prevalent in crops such as basil, cauliflower, endive, escarole, and radicchio, particularly when these are rotated with infected lettuce [10]. 

Disease incidence was estimated using visual zigzag sampling approach, which may introduce sampling bias due to non-random field coverage. In fields with low disease incidence, spatial heterogeneity and aggregated symptom distribution may lead to under- or overestimation of the disease incidence. However, the zigzag approach was chosen to balance field coverage and practical feasibility and is commonly used in large-scale field assessments.

All studied isolates formed typical *Sclerotinia* colonies, producing black sclerotia, either large or small, consistent with the morphological descriptions of *S. sclerotiorum* and *S. minor*, respectively [44,45]. Based on sclerotial size, a reliable morphological criterion for distinguishing the two species [46], 55.1% of isolates derived in the present study were identified as *S. sclerotiorum* and 44.9% as *S. minor*. *S. nivalis*, a third species reported as a lettuce pathogen [11], was not detected. *Sclerotinia* spp. were detected in 8 districts across Serbia, with *S. sclerotiorum* being more frequently isolated. These findings align with those of Kim and Cho [45], who reported a lower isolation frequency of *S. minor* compared to *S. sclerotiorum* in surveyed locations in South Korea. *S. sclerotiorum* has also been published as a dominant species causing lettuce drop in the San Joaquin Valley in California, the USA [47], and in Norway [48]. Our results represent one of the first comprehensive studies on the distribution and prevalence of *Sclerotinia* species on lettuce in Europe. They are also among the few studies available worldwide. This significance is further accentuated by the considerable lack of recent data from most European countries, highlighting the need for sustained and expanded research in this field.

Almost all *S. sclerotiorum* isolates exhibited white or whitish, fluffy aerial mycelium. Only one grey-colored *S. sclerotiorum* isolate was identified. Similarly, *S. minor* isolates mostly formed whitish cottony mycelium, with no darkly pigmented isolates observed. Morphological diversity among *S. sclerotiorum* isolates has been documented across various crops [49,50,51,52,53,54]. Variability in colony color and growth patterns has been reported in isolates from Bangladesh [55], northeastern India [52], and Brazil [56]. Garg et al. [57] were the first to find three darkly pigmented isolates from Australia. Abreu and Souza [58] described Brazilian isolates ranging in color from white and beige to brown and black. In contrast to mycelial appearance, sclerotial size is a widely accepted morphological criterion for distinguishing between *S. sclerotiorum* and *S. minor*. In our study, we observed that the ranges of the average sclerotial size of the two species overlapped. Despite this overlap, the distinction between species remains reliable when considering the overall distribution of sclerotial sizes and their number per Petri plate. Therefore, species identification should take into account not only individual sclerotial size but also the typical size range and the abundance pattern, both of which support clear differentiation between the two species.

Morphological characteristics may provide reliable and accurate *Sclerotina* species discrimination if sclerotia are present. However, molecular methods ensure the most precise and fastest detection and characterization. Different molecular methods have been developed to discriminate *Sclerotinia* species [24,59,60,61,62]. The primers and multiplex PCR protocol described by Abd-Elmagid et al. [24] were tested in this study and confirmed as a fast, sensitive, reliable, and convenient diagnostic technique that successfully complemented the morphological identification of all *Sclerotinia* spp. isolates derived in this study. Molecular analysis proved that the ITS region is highly conserved for *Sclerotinia* species, with only a few nucleotide differences between the studied species. Phylogenetic analysis of 20 Serbian isolates confirmed a closer relationship of *S. sclerotiorum* with *S. nivalis* and *S. trifoliorum* than with *S. minor*, which is consistent with the previous findings [28,61].

The population structure of *S. sclerotiorum*, based on a comprehensive haplotype analysis, showed substantial uniformity and the presence of one dominant haplotype [29]. Before this study, there were scarce data on the population structure of *S. minor* at the global level. Several studies on the mycelial compatibility of *S. minor* have determined genetic diversity in the population, including the identification of 8 mycelial compatible groups (MCGs) among 95 isolates from lettuce and weeds in China [63] and 23 MCGs among isolates from lettuce in California, the USA [47]. Regardless of certain constraints of the conservative nature of the ITS region in *Sclerotinia* spp. in general, and in *S. minor,* our analysis of all available sequences of the worldwide population of *S. minor* implied a clear pattern and the presence of 9 haplotypes arranged in a star-shaped network. All *S. minor* sequences from Serbia were uniform, and all belonged to the dominant Hap2 haplotype. The central position of Hap2 suggests that it could be an ancestral haplotype or a haplotype with higher reproductive success. The fact that all other haplotypes were linked to Hap2 may indicate a clear diversification pattern and suggest recent population expansion or positive selection. Genotype characterization of the field population of *S. minor* [64] showed similar results and the presence of one dominant genotype comprising the largest number of isolates. The haplotype diversity and network analyzed in this study, in conjunction with the geography of origin and host plants, revealed that the most frequently detected haplotypes were Hap2 and Hap1 (comprising 69 and 26 isolates, respectively), detected on five and four continents, respectively, and pathogenic to 15 and 3 host plant species, respectively. This is consistent with previous descriptions that *S. minor* has a limited host range [10,63,65]. Although isolates from Hap2 are pathogenic to 15 different host plants, the majority are associated with a single family, Asteraceae, suggesting later diversification and adaptation to new hosts. The expansion of the host range likely reflects multiple factors involved in the co-evolution of pathogens and host plants [66], as has also been demonstrated for fungi in the Sclerotiniaceae [67].

*Sclerotinia* species are well-known necrotrophic plant pathogens that can effectively induce cell death in host plant tissues. They produce numerous lytic enzymes, including pectinases, cellulases, hemicellulases, and proteases, which aid in colonization and cause the breakdown of host cell walls. Alongside cell wall-degrading enzymes, oxalic acid, considered an essential virulence factor for *Sclerotinia* species, plays a crucial role in the infection process by suppressing host defense responses and inducing programmed cell death in plant cells [15,16,68]. This phytotoxin produced by the fungus serves multiple functions, including the disruption of plant cell membranes, inactivation of enzymes involved in plant defense mechanisms, and the manipulation of host signaling pathways to create a favorable environment for fungal colonization and infection [17,69,70]. Various studies demonstrated that higher oxalic acid-producing isolates were more virulent than those of low-producing isolates [17,71,72,73]; however, in the study of Li et al. [12], a correlation between oxalic acid production and pathogenicity was not found. In the present study, we did not observe a statistically significant correlation between oxalic acid production and virulence in either of the two investigated species. For *S. sclerotiorum*, the correlation was weakly positive, while for *S. minor*, it was weakly negative; however, in both cases, the correlations were not statistically significant. It is important to emphasize that the virulence in our study was assessed using detached leaf assays, which provide a standardized and controlled environment for evaluating isolate aggressiveness. While these assays allow a reliable comparison of relative pathogenic potential among isolates, they may not capture the full complexity of field conditions, including environmental variability and host responses. Therefore, the observed differences in virulence could be interpreted as relative aggressiveness under controlled conditions, rather than as absolute measures of field virulence. These findings suggest that although oxalic acid is an established virulence-associated metabolite in *S. sclerotiorum*, it does not fully account for virulence variation among isolates, and its contribution appears even less pronounced in *S. minor*. The contrasting tendencies between species likely reflect differences in pathogenic strategies and the multifactorial nature of virulence, including factors such as cell wall-degrading enzymes, pH modulation, effector secretion [15,70,73,74,75], and potentially, the timing and localization of oxalic acid production. Therefore, oxalic acid should be considered one component of a broader virulence repertoire. Future work integrating multiple biochemical and molecular determinants is warranted to fully resolve pathogenic variability. Additional studies incorporating larger sample sizes and assays under conditions that better reflect the natural environment are also required to elucidate the relationship between oxalic acid production and virulence in these species.

Microorganisms are continuously exposed to a variety of abiotic stressors, including ultraviolet radiation, temperature fluctuations, disruptions in osmotic homeostasis, and oxidative stress [18]. Understanding how they respond to such challenging conditions is essential not only for elucidating the ecophysiology of microorganisms in diverse natural habitats but also for advancing food safety and predicting pathogen virulence [76]. For instance, high concentrations of NaCl induce osmotic stress, which can significantly inhibit the mycelial growth of many fungi [18]. Araújo et al. [77] assessed osmotic susceptibility across 70 fungal strains representing 40 ecologically diverse species and found that fungi exhibiting a trophic dependency on hosts were more susceptible to high osmotic pressure. The results of the present study showed that *S. minor* was significantly more susceptible to increased osmotic pressure compared to *S. sclerotiorum*. This finding may indicate a higher level of physiological adaptability or osmotic and saline stress tolerance in *S. sclerotiorum*. This could partly explain its wider distribution and prevalence as a plant pathogen globally. In contrast, *S. minor* exhibited higher susceptibility to osmotic stress, suggesting potential limitations in its ecological adaptability under conditions of environmental stress. Given that osmotic stress can be a factor in soil environments, especially under drought or saline conditions, these physiological differences may affect species survival and virulence in the field. Therefore, further investigation of their environmental resilience, particularly that of *S. minor,* is warranted, given its recent emergence in this region [21].

## 5. Conclusions

This study provides new insights into lettuce drop disease in Serbia. Through pathogenicity and virulence assays, morphological characterization, and molecular analysis, *S. sclerotiorum* and *S. minor* were confirmed as the causal agents of the disease. A statistically significant difference between the two species was detected in growth rate, virulence, oxalic acid production, and osmotic susceptibility. No significant relationship between oxalic acid production and virulence was identified, further highlighting the multifactorial nature of pathogenicity in *Sclerotinia* species. Given the scarcity of up-to-date data on *Sclerotinia* populations in Europe, these findings fill a critical knowledge gap and provide a foundation for the development of more effective and targeted management strategies. They also serve as an essential reference for future research on lettuce and other horticultural crops.

## Figures and Tables

**Table 1:** Isolates of *Sclerotinia* spp. included in phylogenetic analyses.

Species	Acc No	Isolate	Host	Origin	Submitted	Reference
*Sclerotinia minor*	JF279880	62907	*Tanacetum* sp.	Australia	2011	[28]
	AB516661	MAFF 238173	-	Japan	2009	[28]
	JF279879	45903A	-	Australia	2011	[28]
	JF279877	45802A	-	Australia	2011	[28]
	OL423632	15-2	*Lactuca sativa*	Serbia	2021	[21]
	PQ489371	Sm 11-4	*Lactuca sativa*	Serbia	2021	In this study
	PQ489372	Sm 11-9a	*Lactuca sativa*	Serbia	2021	In this study
	PQ489373	Sm 11-10	*Lactuca sativa*	Serbia	2021	In this study
	PQ489374	Sm 13-2	*Lactuca sativa*	Serbia	2021	In this study
	PQ489375	Sm 13-5	*Lactuca sativa*	Serbia	2021	In this study
	PQ489376	Sm 15-3	*Lactuca sativa*	Serbia	2022	In this study
	PQ489377	Sm 15-5	*Lactuca sativa*	Serbia	2022	In this study
	PQ489378	Sm 18-3	*Lactuca sativa*	Serbia	2022	In this study
	PQ489379	Sm 18-4	*Lactuca sativa*	Serbia	2022	In this study
	PQ489380	Sm 18-5	*Lactuca sativa*	Serbia	2022	In this study
*Sclerotinia sclerotiorum*	KX184720	2	*Brassica oleracea* var. *capitata*	Sri Lanka	2017	[28]
	MG249967	SSC2JHU	*Gossypium hirsutum*	USA	2017	[28]
	AB233346	MAFF 306676	*Vaccinium corymbosum*	Japan	2007	[28]
	KY073613	16-042	*Capsella bursa-pastoris*	Korea	2016	[28]
	KP340898	SS-BO-SC	*Brassica oleracea* var. *capitata*	New Mexico	2015	[28]
	JN013184	-	*Aquilegia flabellata*	Italy	2011	[28]
	KF859932	DAOM:241671	-	Canada	2013	[28]
	DQ329537	B23	-	Alaska	2006	[28]
	HQ833450	Ms85	*Morus alba*	China	2010	[28]
	KY750530	SQC-000	*Oenanthe javanica*	China	2017	[28]
	KY073614	16-119	*Cucumis sativus*	Korea	2016	[28]
	FJ810516	ATCC:MYA-4521	-	USA	2009	[28]
	KT224645	Ss1212HA	*Trifolium ambiguum*	Poland	2015	[28]
	KJ614564	JBARES2014A	*Allium tuberosum*	Korea	2014	[28]
	AB937095	MuRa-103	*Brassica napa*	Japan	2014	[28]
	KJ744364	PM27	*Beta vulgaris*	Germany	2014	[28]
	KY073612	15-030	*Lactuca sativa*	Korea	2016	[28]
	KF791510	PSHB1	*Lablab purpureus*	Bangladesh	2013	[28]
	PP179050	B	*Brassica oleracea* var. *capitata*	Serbia	2025	[29]
	PP179051	B1	*Brassica oleracea* var. *capitata*	Serbia	2025	[29]
	PP179052	B2	*Brassica oleracea* var. *capitata*	Serbia	2025	[29]
	PP179053	B3	*Brassica oleracea* var. *capitata*	Serbia	2025	[29]
	PP179054	M	*Brassica oleracea* var. *capitata*	Serbia	2025	[29]
	PP179057	M1	*Brassica oleracea* var. *capitata*	Serbia	2025	[29]
	PP179055	M2	*Brassica oleracea* var. *capitata*	Serbia	2025	[29]
	PP179056	M3	*Brassica oleracea* var. *capitata*	Serbia	2025	[29]
	PP179058	M4	*Brassica oleracea* var. *capitata*	Serbia	2025	[29]
	PP179059	M5	*Brassica oleracea* var. *capitata*	Serbia	2025	[29]
	PP179060	M6	*Brassica oleracea* var. *capitata*	Serbia	2025	[29]
	PP179061	M7	*Brassica oleracea* var. *capitata*	Serbia	2025	[29]
	PP177498	SC	*Helianthus annuus*	Serbia	2025	[29]
	PQ489358	Ss 17-7	*Lactuca sativa*	Serbia	2022	In this study
	PQ489359	Ss 18-10	*Lactuca sativa*	Serbia	2022	In this study
	PQ489360	Ss 18-11	*Lactuca sativa*	Serbia	2022	In this study
	PQ489361	Ss 20-1	*Lactuca sativa*	Serbia	2022	In this study
	PQ489362	Ss 20-3	*Lactuca sativa*	Serbia	2022	In this study
	PQ489363	Ss 21-1	*Lactuca sativa*	Serbia	2022	In this study
	PQ489364	Ss 21-2	*Lactuca sativa*	Serbia	2022	In this study
	PQ489365	Ss 22-11	*Lactuca sativa*	Serbia	2022	In this study
	PQ489366	Ss 22-14	*Lactuca sativa*	Serbia	2022	In this study
	PQ489367	Ss 23	*Lactuca sativa*	Serbia	2022	In this study
*Sclerotinia trifoliorum*	KT819299	TN Sc10101	*Trigonella foenum-graecum*	Tunisia	2015	[28]
	KT224652	St03TP	*Trifolium pretense*	Poland	2015	[28]
	KT224651	St02TP	*Trifolium pretense*	Poland	2015	[28]
	KT986229	St1813TP	*Trifolium pretense*	Poland	2015	[28]
*Sclerotinia nivalis*	JX294862	SN110812	*Atractylodes japonica*	China	2012	[30]
	PV973881	Sm8	*Daucus carota* subsp. *sativus*	Russia	2023	[31]
*Hypocrea lixii*	FJ861393	2S12A	-	USA	2009	[28]

**Table 2:** Lettuce drop incidence in different districts in Serbia during 2021 and 2022.

Year	District	Location	Disease Incidence ^1^	No. of Samples	Fungal Species Detected ^2^
2021	South Banat	Glogonj 1	10%	7	*Phoma* sp. (1) ^3^ *Plectosphaerella* spp. (3)
		Glogonj 2	5%	9	*Fusarium* spp. (2) *Plectosphaerella* sp. (1)
		Glogonj 4	2–5%	4	*Pythium* sp. (1)
		Glogonj 5	2%	3	*Alternaria* sp. (1)
	West Bačka	Bački Brestovac	2%	6	*Fusarium* sp. (1)
	Jablanica	Navalin 1	5%	5	*Alternaria* sp. (1)
		Navalin 2	10%	3	*Verticillium* spp. (2) *Alternaria* sp. (1)
		Navalin 4	2%	4	*Fusarium* sp. (1)
		Donja Lokošnica	2%	3	*Plectosphaerella* spp. (3)
		Priboj	5–10%	4	*Verticillium* spp. (2) *Alternaria* sp. (1)
	Zlatibor	Potočanje	55%	8	*Sclerotinia minor* (5) *Fusarium* sp. (1)
2022	South Banat	Opovo 1	1%	7	*Plectosphaerella* sp. (1)
		Opovo 2	1%	4	*Plectosphaerella* sp. (1)
	Mačva	Mrđenovac 1	2%	1	*Fusarium* sp. (1)
		Mrđenovac 2	5%	4	*Fusarium* spp. (2)
		Žabar	5%	1	*Plectosphaerella* sp. (1)
	Moravica	Trbušani 1	30%	20	*Sclerotinia minor* (19) *Botrytis* spp. (3)
		Trbušani 3	15%	7	*Sclerotinia minor* (3) *Botrytis* sp. (1)
	Nišava	Merošina 1	25%	3	*Fusarium* spp. (3)
		Merošina 2	1%	1	*Fusarium* sp. (1)
	Pirot	Bela Palanka	35%	25	*Sclerotinia sclerotiorum* (10) *Botrytis* sp. (1)
	Toplica	Blace	20%	23	*Sclerotinia sclerotiorum* (5) *Sclerotinia minor* (8) *Botrytis* sp. (1)
	Jablanica	Lebane 2	30%	12	*Sclerotinia sclerotiorum* (10) *Botrytis* spp. (3)
	Podunavlje	Smederevska Palanka	15%	7	*Sclerotinia sclerotiorum* (5) *Botrytis* sp. (2)
	Srem	Irig	40%	12	*Sclerotinia sclerotiorum* (11) *Botrytis* sp. (1)
		Kraljevci	2%	1	*Sclerotinia sclerotiorum* (1)
	South Bačka	Veternik	15%	1	*Sclerotinia sclerotiorum* (1) *Botrytis* spp. (3)

^1^ Average disease incidence estimated by walking through the crop in a zigzag pattern and randomly rating 100 plants in three replicates. ^2^ Isolated pathogenic species identified to the genus level based on colony morphology and ITS sequencing. ^3^ The numbers in parentheses represent the number of isolates obtained.

**Table 3:** Cultural features of *Sclerotinia* spp. isolates on potato dextrose agar at 25 °C.

Species	Isolate	Colony Color	Relative Density and Appearance of Aerial Mycelium	Dark Pigment Synthesis	Timing of Formation of Sclerotia (Day)	Arrangement of Sclerotia	Average No of Sclerotia ± SD/Plate	Average Diameter of Sclerotia (mm)
*Sclerotinia sclerotiorum*	Ss 17-7	Snow white	Woolly- floccose, dense	-	8	Edge and middle ring	12.7 ± 8.1	1.67–5.17
	Ss 18-10	Grey	Floccose, dense	+	9	Scattered all around	65.0 ± 8.7	0.67–2.17
	Ss 18-11	Whitish	Floccose, low dense	-	7	Edge and middle ring	20.0 ± 13.0	0.83–3.33
	Ss 20-1	Whitish	Floccose, very dense	-	7	Scattered all around	36.3 ± 5.1	1.17–3.33
	Ss 20-3	Whitish	Floccose, dense	+	9	Edge and middle ring	24.0 ± 7.9	1.00–2.83
	Ss 21-1	Snow white	Woolly, dense	-	8	Edge and middle ring	8.7 ± 1.5	2.33–5.83
	Ss 21-2	White	Floccose, low dense	-	8	Scattered all around	21.7 ± 6.5	1.50–4.33
	Ss 22-11	White	Floccose, low dense	-	8	Middle ring	2.3 ± 0.6	3.67–5.00
	Ss 22-14	White	Woolly- floccose, low dense	-	7	Edge and middle ring	20.7 ± 3.5	0.90–4.00
	Ss 23	Whitish	Floccose, dense	-	8	Scattered all around	14.3 ± 3.1	1.67–3.33
*Sclerotinia minor*	Sm 11-4	Whitish	Floccose, dense	-	6	Scattered all around	373.3 ± 23.1	0.50–1.50
	Sm 11-9a	Snow white	Woolly, dense to very dense	-	8	Scattered all around	153.3 ± 30.6	0.40–2.33
	Sm 11-10	Whitish	Cottony- floccose, low dense	-	8	Scattered all around	120.0 ± 20.0	0.50–1.75
	Sm 13-2	Whitish	Floccose, low dense to dense	-	6	Scattered all around	200.0 ± 40.0	0.50–2.00
	Sm 13-5	Whitish	Floccose, low dense	-	8	Scattered all around	186.7 ± 61.1	0.50–1.50
	Sm 15-3	Whitish	Floccose, dense	-	6	Scattered all around	140.0 ± 10.0	1.00–2.00
	Sm 15-5	White	Floccose, low dense to dense	-	9	Scattered all around	46.7 ± 11.5	1.00–2.50
	Sm 18-3	Whitish	Cottony- floccose, very dense	-	8	Scattered all around	95.0 ± 39.7	0.67–1.83
	Sm 18-4	Whitish	Cottony, dense	-	8	Edge ring	108.3 ± 20.2	0.50–1.83
	Sm 18-5	Whitish	Cottony, dense	-	9	Edge ring	11.7 ± 5.9	0.20–0.50

## Data Availability

The original contributions presented in this study are included in the article. Further inquiries can be directed to the corresponding author.

## Abbreviations

The following abbreviations are used in this manuscript:

bp	Base pair
DNA	Deoxyribonucleic Acid
Hap	Haplotype
ITS	Internal Transcribed Spacer
PCR	Polymerase Chain Reaction
PDA	Potato Dextrose Agar
nt	Nucleotide
SD	Standard Deviation
